# Outbreak of invasive Group A streptococcus disease in a nursing home in Ireland in February 2023 caused by *emm* type 18

**DOI:** 10.2807/1560-7917.ES.2024.29.17.2300609

**Published:** 2024-04-25

**Authors:** Ciara Carroll, Mary Meehan, Roisin Connolly, Jayne Prendergast, Colette Magnone, Aine Meehan, Chantal Migone, Keith Ian Quintyne, Caroline Carpenter, Helen Byrne, Robert Cunney, Paul Mullane

**Affiliations:** 1 Public Health HSE Dublin and North East, Dr Steeven’s Hospital, Dublin & Kells Business Park, Kells, Ireland; 2 Irish Meningitis and Sepsis Reference Laboratory, Children’s Health Ireland at Temple Street, Dublin, Ireland; 3 Our Lady of Lourdes Hospital, Drogheda, Ireland; 4 National Immunisation Office, Manor Street Business Park, Dublin, Ireland; 5 Health Protection Surveillance Centre, Dublin, Ireland

**Keywords:** invasive Group A streptococcus, outbreak, nursing home

## Abstract

An out-of-season increase in cases of invasive Group A streptococcus (iGAS) was observed in Ireland between October 2022 and August 2023. We describe the management of an iGAS outbreak involving three nursing home residents in Ireland in early 2023. A regional Department of Public Health was notified of an iGAS case in a nursing home resident in January 2023. When two further cases among residents were notified 7 days later, an outbreak was declared. Surveillance for GAS/iGAS infection in residents and staff was undertaken. The site was visited to provide infection prevention and control (IPC) support. Isolates were *emm* typed. A total of 38 residents and 29 staff in contact with resident cases were provided with antibiotic chemoprophylaxis. Seven additional staff with no direct resident contact also received chemoprophylaxis after finding one probable localised GAS infection among them. No more iGAS cases subsequently occurred. Site visit recommendations included advice on terminal cleaning and cleaning of shared equipment, as well as strengthening staff education on hand hygiene and masking. All isolates were of* emm* subtype 18.12, a subtype not previously detected in Ireland. Key outbreak control measures were rapid delivery of IPC support and chemoprophylaxis. *Emm*18 is infrequently associated with GAS infections.

**Key public health message**

**What did you want to address in this study and why?**

Invasive Group A streptococcus (iGAS) can cause life-threatening diseases including necrotising fasciitis and toxic shock syndrome. In nursing homes, where iGAS outbreaks are associated with high case fatality rates, the rapid implementation of outbreak control measures is critical. In this report, we discuss the response to the first known outbreak of iGAS in a nursing home in Ireland, involving three microbiologically linked cases.

**What have we learnt from this study?**

All cases had been notified within a 7-day period and were affected by an iGAS strain of the *emm* sequence type 18. The rapid delivery of infection prevention and control (IPC) support in conjunction with mass chemoprophylaxis helped to control this outbreak, with no further cases detected following the implementation of these measures. There is an ongoing need to reinforce the importance of IPC measures in all settings with vulnerable residents.

**What are the implications of your findings for public health?**

The control of iGAS outbreaks in nursing homes is dependent on the rapid identification of cases, scrupulous IPC measures and the appropriate delivery of chemoprophylaxis guided by public health risk assessment. This report adds to the limited evidence base on the epidemiology of *emm* sequence type 18, which is uncommon in Ireland and elsewhere in Europe.

## Background

*Streptococcus pyogenes* (Group A streptococcus, GAS) is a common cause of community-acquired skin, soft tissue and respiratory tract infections, with rarer invasive infection causing life-threatening conditions such as streptococcal toxic shock syndrome and necrotising fasciitis [1]. Globally, there are ca 650,000 cases and over 160,000 deaths attributed to invasive Group A streptococcus (iGAS) disease each year [2]. GAS is a highly contagious pathogen which can be transmitted through respiratory droplets and direct contact with infected people or contaminated surfaces [3]. In December 2022, the World Health Organization reported a multi-country surge in the number of cases of iGAS disease in Europe and the United States (US) [4]. In Ireland, an unseasonal increase in iGAS cases was observed in late 2022 and early 2023, with the largest increase in cases observed in teenagers and children aged below 18 years and most cases caused by *emm*1 (47%) or *emm*12 (25%) sequence types [5]. In this outbreak report, we discuss an iGAS outbreak in a nursing home in Ireland caused by the uncommon *emm* sequence type 18. 

### Outbreak detection

In January 2023 (Day 0), a regional Department of Public Health (DPH) was notified of a case of iGAS in an elderly nursing home resident (Case A) who had been admitted to an acute hospital with cellulitis. GAS was isolated from blood cultures and from a limb swab, both taken on admission to the acute hospital 2 days previously. Initial risk assessment of residents and staff in the nursing home was performed following United Kingdom Health Security Agency (UKHSA) guidelines [6]. UKHSA guidelines were used as Irish guidelines specific to nursing home settings were not available at the time of the outbreak. There were ca 60 residents and 40 staff in the facility, with up to 10 staff involved in providing care to the index case. No residents or staff with symptoms suggestive of GAS infection were identified. As the index case had a single room, there were no household-type contacts identified who met the criteria for considering chemoprophylaxis. A “Warn and Inform” letter was issued to the person in charge (PIC) in the nursing home, with advice to be vigilant regarding other potential cases among residents and staff.

A week later (Day 7), the regional DPH received notification from the same acute hospital of two further cases of iGAS in residents of the same facility. The second case (Case B) had been admitted to hospital 3 days after the notification of Case A. Case B had developed a facial rash 5 days before the notification of the index case and was transferred to hospital on Day 3 when the rash spread and pharyngitis and fever (i.e. temperature of over 38.0˚C) developed. GAS was isolated from blood cultures and from a swab of a nasal lesion taken on admission. The third case (Case C) had been discharged from the acute hospital to the nursing home on Day 3 following hospitalisation for a non-GAS related issue. They were re-admitted to hospital on Day 6 with thrombophlebitis at the site of a previous peripheral venous catheter. GAS was isolated from blood cultures taken on admission.

An outbreak was declared, and an outbreak control team (OCT) urgently convened on Day 7. The OCT had a specialist in public health medicine in the DPH as its chair, with the nursing home PIC and general practitioner (GP), as well as a clinical microbiologist and infection prevention and control (IPC) team members from the acute hospital, and members of the regional DPH team in attendance.

## Methods

### Case detection

Following the notification of Cases B and C to the DPH, a review of iGAS cases notified in the region in the previous 6 months was undertaken to identify other cases who may be linked to the nursing home. Vigilance for signs and symptoms of GAS infection was advised for all residents and staff. Nursing home residents and the staff caring for them were advised to report any symptoms suggestive of GAS infection so that these could be assessed by the nursing home GP. Staff were advised to attend their own GP if they developed any symptoms. DPH staff also interviewed all staff contacts of the resident cases to check for any symptoms which may be due to GAS or iGAS infection. A review of deaths in the nursing home in the 4 weeks prior to symptom onset in the index case was undertaken to determine if any of these may be related to GAS.

### Case definition

A case definition was agreed by the OCT as follows:

• A confirmed case was a laboratory confirmed case of iGAS in a person (staff or patient) linked (i.e. directly or indirectly) to the nursing home from the date of notification of Case A. Laboratory confirmation refers to the isolation of GAS by culture or molecular methods (such as PCR), from a normally sterile body site, in keeping with the Health Protection Surveillance Centre case definition [7].

• A probable case was any symptomatic case (staff or patient), with epidemiological links to a confirmed case linked to the nursing home from the date of notification of Case A. Symptoms suggestive of iGAS infection disease included high fever, severe muscle aches, localised muscle tenderness, increasing pain, swelling and redness at site of wound, unexplained diarrhoea or vomiting [8]. Symptoms suggestive of non-invasive GAS infection included sore throat, fever, minor skin infections and scarlatiniform rash [8].

### Microbiological investigation

Blood cultures (BacTAlert, bioMérieux) were obtained from the three nursing home residents with symptoms of GAS on admission to the acute hospital. Charcoal swabs (Copan Transystem) were obtained from relevant sites for those with symptoms suggestive of GAS infection. Isolates were identified by Matrix Assisted Laser Desorption Ionization-Time of Flight Mass Spectrometry (MALDI-TOF MS; Bruker). Susceptibility testing was performed on Vitek 2 (bioMérieux) according to European Committee on Antimicrobial Susceptibility Testing methodology [9].

Following the isolation of GAS from blood cultures for the three confirmed cases, isolates were sent to the Irish Meningitis and Sepsis Reference Laboratory (IMSRL) in Dublin for *emm* sequence typing. *Emm* sequence typing was performed as previously described [10]. Genomic DNA was sequenced on an Illumina 2500 HiSeq sequencer (Wellcome Trust centre for Human Genetics, University of Oxford, UK) using 150 bp paired-end reads. Single nt polymorphisms (SNPs) were called using Snippy (version 4.6.0, https://github.com/tseemann/snippy). Genealogies Unbiased By recomBinations In Nt Sequences (Gubbins; version 2.4.1) was used to remove regions of high SNP density due to suspected recombination [11].

Consideration was given by the OCT to swabbing all contacts of the cases among residents and staff in the nursing home to allow for a targeted approach to chemoprophylaxis. However, the OCT decided not to swab contacts unless they had symptoms suggestive of GAS infection. This was to prevent delays in the implementation of control measures while awaiting results and to ensure that GAS colonisation in anatomical sites which are not routinely swabbed was not overlooked. As no epidemiologically linked shared equipment or facilities were identified, no environmental sources were identified for microbiological investigation.

### Site visit

A site visit was undertaken by members of the DPH team on Day 9 to assess iGAS outbreak control measures, cleaning and disinfection, and the management of on-site facilities. Meetings were held with nursing home management, followed by a walk-through of the facility. Governance arrangements, education and training and IPC audits were reviewed, followed by an assessment of environmental cleaning and disinfection.

## Results

### Case overview

In total, there were three confirmed cases of iGAS identified during this outbreak, summarised in the Table. The timeline of events for the outbreak is illustrated in the Figure. Of the three cases, two had underlying conditions which increased their risk of iGAS, namely diabetes and a non-healing lower limb ulcer, and the use of immunosuppressant medications. All three cases resided in the main building of the facility, with no cases identified in the adjacent unit which was completely separate for residents, with no access to the main building and discrete dining and communal areas. However, staff working in the adjacent unit shared changing facilities with staff working in the main building.

**Table:** Summary of confirmed invasive Group A streptococcus cases linked to an outbreak in a nursing home, Ireland, 2023 (n = 3 cases)

Case	Age category in years	Symptom onset^a^	Day of blood cultures^a^	Day notified^a^	Clinical presentation
A	≥ 75	Day − 3	Day 2	Day 0	Cellulitis
B	< 75	Day − 5	Day 3	Day 7	Pharyngitis, impetigo
C	≥ 75	Day 6	Day 6	Day 7	Cellulitis, thrombophlebitis

^a^ Days are numbered relative to the day the index case (case A) was notified (Day 0).

**Figure:**
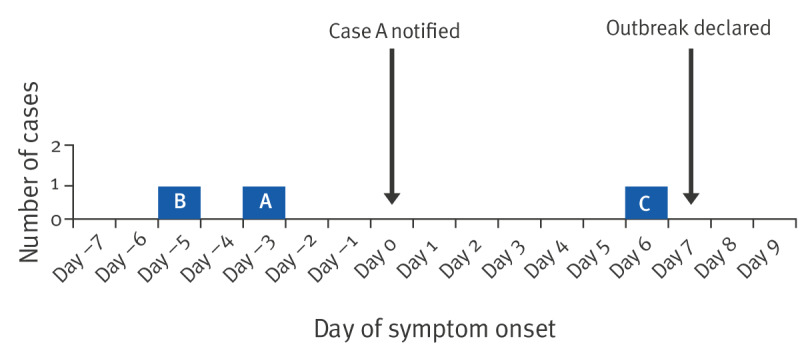
Distribution according to timing of symptom onset of confirmed invasive Group A streptococcus cases linked to an outbreak in a nursing home, Ireland, 2023 (n = 3 cases)

Case A was the index case for this outbreak.

The mode of transmission between cases was not established. Given the small number of cases linked with the outbreak and the absence of further cases arising following the implementation of outbreak control measures, no analytical study was carried out.

Of the three confirmed cases, one case developed a complication and died in hospital within a month of admission. Although this death was not directly attributed to iGAS infection, iGAS was reported as an antecedent cause of death. The other two cases recovered and were discharged back to the nursing home.

DPH staff received reports of illness among five further residents of the nursing home, including a resident who was admitted to an acute hospital with a non-GAS related issue. Of the other four unwell residents, three did not have symptoms suggestive of non-invasive or iGAS infection. A facial swab was performed for the fourth unwell resident, who resided in the unit adjacent to the main building and developed a bilateral facial rash on Day 16; GAS was not isolated. The review of three recent deaths did not identify any deaths suspected to be due to iGAS.

A member of support staff who had no direct contact with residents was suspected to have non-invasive GAS infection after developing pharyngitis on Day 9 and was commenced on amoxicillin by their GP. No microbiological tests were performed, so the diagnosis of GAS infection was not confirmed. There were no other reports of illness among staff.

### Microbiological results

Bloodstream isolates from all three resident cases were identified as *S. pyogenes*. *S. pyogenes* was also isolated from infected skin lesions from Case A and Case B. All isolates were susceptible to all agents tested, including penicillin, erythromycin, clindamycin and tetracycline.

All isolates were of *emm*18.12 sequence subtype, an *emm* subtype not previously detected in Ireland. Interrogation of PubMLST.org revealed that all isolates were multilocus sequence type 535. Genomic core SNP analysis was limited due to the fact that there were no *emm*18.12 isolates in the Irish culture collection other than the outbreak isolates and no publicly available sequences. SNP analysis was therefore performed by sequence comparison of the three *emm*18.12 outbreak isolates with four sporadic *emm*18 isolates in the Irish culture collection (consisting of two *emm*18 isolates and one of each of *emm*18.29 and *emm*18.31; data available from 2012 onwards). This revealed that the core genomes of the outbreak-associated isolates differed by 3–5 SNPs compared with a minimum of 23 SNPs between genomes of the other four sporadic *emm*18 isolates and a reference genome (MGAS8232; GenBank accession number: AE009949).

### Findings from site visit

At the time of the outbreak, all rooms were at single occupancy. Appropriate education and training on IPC measures had been provided to all staff. Hand hygiene and environmental audits had been performed in January 2023 and were completed again on Day 8 ahead of the site visit, with no issues identified. At the site visit, all areas were visibly clean. Some soft furnishings were noted, along with shared items in common areas which would be difficult to clean, such as books. Touch screens and shared computers were not on a cleaning schedule and no cleaning products were available for these. A missed opportunity for hand hygiene, a member of staff not adhering to Bare Below Elbows (BBE) and incorrect wearing of a face mask were also observed. Advice was provided on terminal cleaning, cleaning of shared equipment, and on the need for ongoing education on hand hygiene and Personal Protective Equipment (PPE). It was recommended that soft furnishings be replaced with cleanable chairs and couches, and to consider removing shared items such as books during the outbreak. No epidemiologically linked equipment or facilities requiring environmental sampling were identified.

### Outbreak control measures

Following the first meeting of the OCT on Day 7, admissions and transfers to the main part of the nursing home building were suspended, with visitors limited to nominated support persons as defined in Irish guidelines on the management of COVID-19, influenza and other respiratory infections in nursing homes [12]. Staff movements were restricted to as few units within the nursing home as was safely practicable for resident care. An alert was issued to residents, residents’ next-of-kin and staff regarding the declaration of the outbreak and the recommendations of the OCT. Vigilance for symptoms suggestive of non-invasive GAS infection or iGAS was recommended for staff and residents, with a low threshold for informing Public Health of any concerns. The GP for the nursing home was available to review cases. Screening of staff and residents in the facility was considered, but chemoprophylaxis was recommended by the OCT to prevent delays in eliminating GAS carriage in the context of vulnerable residents with high care needs. Chemoprophylaxis was recommended for all residents of the main building of the facility and for staff who had direct clinical contact with resident cases in the 7 days before the earliest date of symptom onset. Based on recommendations from Clinical Microbiology, and in accordance with national guidelines [13], amoxicillin was the first line agent offered, with clarithromycin offered as a second-line agent where required. Chemoprophylaxis was initially provided to 38 residents and 27 staff.

A second meeting of the OCT was convened on Day 10, where chemoprophylaxis was recommended for two additional members of non-clinical staff who were identified as having close contact with cases. A third OCT meeting was held on Day 16, where it was identified that a member of support staff with no direct patient contact had symptoms suggestive of non-invasive GAS infection and was managed as a probable case. Following risk assessment, chemoprophylaxis was offered to the probable case and six other members of staff who had been in contact with them. As no new iGAS cases were identified by Day 20, the restrictions on visitors, admissions and transfers were lifted on this date, based on UKHSA guidance [6]. The outbreak was declared over at the final OCT meeting on Day 23, with advice for the facility to remain vigilant regarding symptoms of GAS infection among staff and residents until 30 days had elapsed since the latest date of symptom onset among the confirmed cases. No residents or staff were reported to develop symptoms during this period.

## Discussion

Although GAS typically causes benign infections such as pharyngitis and impetigo, rarer invasive disease such as sepsis, toxic-shock-like syndrome and necrotising fasciitis is associated with significant morbidity and mortality [14]. A surge in cases of iGAS was reported in the UK [15] and the Netherlands [16] in late 2022, with the largest increase in cases and deaths observed among children. This is similar to the situation in Ireland, where of 152 cases of iGAS notified between 02 October 2022 and 25 February 2023, 37% were in children aged under 18 years [5]. There were 16 iGAS-related deaths during this time, with six occurring in children and 10 in adults aged 18 years and older [5]. In Ireland in 2018, the most recent year before the COVID-19 pandemic where detailed surveillance data have been published, there were 136 cases of iGAS, 24% of which were aged under 18 years and 36% of which were aged 65 years or older, in keeping with previous years [17]. There were eight deaths where iGAS was determined to be the main or a contributory cause of death; the ages of these cases were not reported [17]. Limited surveillance data have been published from 2019 to 2021, though the number of cases notified during these years has been reported, at 108, 43 and 35 cases in 2019, 2020 and 2021 respectively [18]. Suggested explanations for the rise in iGAS cases among children include increased varicella virus circulation and the adverse impact of non-pharmaceutical interventions (NPIs) such as social distancing and face masks during the COVID-19 pandemic on childhood immunity [15,16]. It is unclear if these factors may have a role in the development of iGAS in older patients, such as the cases involved in this outbreak.

The risk of iGAS infection and of iGAS-related deaths is higher among residents of nursing homes than in individuals of the same age residing in the community, with case-fatality rates of 21% and 11% respectively reported for iGAS cases in England between 2009 and 2010 [19], and of 35% and 18% respectively in Minnesota, US between 1995 and 2006 [20]. While transmission of iGAS infection may be increased in nursing homes where elderly residents with multiple co-morbidities are in close contact [21], to our knowledge, no outbreaks of iGAS in nursing homes have been notified in Ireland since the introduction of the Computerised Infectious Disease Reporting (CIDR) system in 2004. In iGAS outbreaks in nursing homes in other countries, the main control measures that have been instituted were delivery of mass or selective chemoprophylaxis, microbiological screening, and enhanced IPC measures [22].

A systematic review on the effectiveness and safety of antibiotic chemoprophylaxis for iGAS contacts included six studies where mass chemoprophylaxis was provided to nursing home residents and staff; no further GAS cases were reported in these studies following the administration of chemoprophylaxis [23]. In previous nursing home outbreaks where screening of staff and residents to determine levels of nasopharyngeal carriage was undertaken, carriage was reported in 6.4% of nursing homes residents and staff in a UK outbreak in 1994 [24], and in 4% of residents and 1.6% of staff in a series of outbreaks in nursing homes in the US between 1988 and 1991 [25]. However, while screening may allow for more targeted delivery of chemoprophylaxis, pooled results from a review of 20 outbreaks in nursing homes did not identify an advantage to selective or mass antibiotic chemoprophylaxis [22].

The increased prevalence and severity of iGAS in nursing home residents has been associated with the increased frequency of risk factors in this population including advanced age, the presence of underlying medical conditions, physical debilitation, and requiring significant nursing assistance [20,26]. All three confirmed iGAS cases linked to this outbreak had at least one of these risk factors. Inadequate IPC practices have been identified in many nursing home outbreak investigations, with improvements in IPC measures often leading to a cessation in further GAS transmission [26]. Environmental swabbing in a prolonged UK outbreak in 1994 identified widespread distribution of GAS in carpets, upholstered furniture and curtains, with the outbreak only controlled after the implementation of cleaning measures [24]. The rapid delivery of IPC advice to the nursing home in this outbreak is thus likely to have played an important role in the control of this outbreak. Finally, appropriate public health management of iGAS cases in nursing homes requires effective disease surveillance and timely communication between nursing, hospitals and public health staff to ensure that the required control measures are instituted promptly [20,26]. Following the notification of the cases linked to this outbreak by hospital clinicians, DPH staff communicated rapidly with the nursing home on notification of the index case to provide guidance. A multidisciplinary OCT was urgently convened when Cases B and C were notified and control measures were swiftly introduced.

There are over 250 GAS *emm* sequence types and 1,500 subtypes documented worldwide to date. The most common *emm* types associated with invasive disease are *emm*1, *emm*3, *emm*12, *emm*28 and *emm*89 [27]. Sequence type *emm*18 is uncommon in Europe and North America but has been associated with pharyngitis outbreaks and the subsequent onset of acute rheumatic fever [28-30]. A relatively high prevalence (9%) of *emm*18 among iGAS was reported one in Italian study, 2003–2005 [28]. It was also prevalent (9%) among isolates collected from healthy school children in Korea in 2002 [31] and was shown to be positively associated with invasive disease in Tunisia in isolates collected between 2000 and 2006 [32]. M protein 18 was included in the 26-valent M protein-based vaccine but not in the later 30-valent vaccine [33]. To date these products remain in development and no vaccines have been licensed to prevent GAS [33].

Prior to this outbreak, *emm*18 had only been detected in four of 1,000 typed iGAS isolates in the IMSRL GAS culture collection (data available from 2012 onwards) including two of 120 iGAS cases in 2014 (*emm*18 and *emm* 18.29), one of 125 iGAS cases in 2016 (*emm* 18.21), one of 102 iGAS cases in 2017 (*emm*18). Subtype *emm* 18.12 had not been previously reported in Ireland [17]. The detection of an unusual subtype was sufficient to confirm a microbiological link between the three patients. Multilocus sequence typing and SNP-based analysis, though limited, confirmed the relatedness of the three isolates. Further analysis of the three genomes revealed that all three isolates possessed the *hasABC* operon encoding for the hyaluronic acid capsule and considered to be an important virulence factor of *emm*18 strains [29,34]. As reported elsewhere for *emm*18 isolates, the three outbreak isolates also harboured the *speA* gene encoding the SpeA superantigen [29]. The *speA* gene, also present in *emm*1 and *emm*3, has been shown to be positively associated with invasive disease [35].

The delivery of IPC advice to the nursing home by the OCT and at the site visit are likely to have played an important role in the control of this outbreak, as previous prolonged outbreaks in nursing home have only been controlled after the implementation of cleaning measures [24]. Following administration of chemoprophylaxis to staff and residents, there were no further cases linked with the facility, suggesting that this was effective in eliminating GAS carriage in staff and residents of the nursing home. Rapid turnaround on *emm* typing results confirmed a microbiological link between cases in addition to the epidemiological link and guided subsequent outbreak control measures.

The delivery of mass chemoprophylaxis without undertaking swabbing for staff and residents of the nursing home precluded the identification of the number of residents and staff who were colonised and potential routes of person-to-person transmission of GAS within the facility. In the context of growing concerns about antimicrobial resistance, offering chemoprophylaxis to seven additional staff who had no direct patient contact and limited contact with the probable case of non-invasive GAS infection may have been an overly conservative approach.

## Conclusion

This report describes an iGAS outbreak in a nursing home in Ireland caused by the uncommon *emm*18 sequence type. To our knowledge, this is the first such outbreak in a nursing home in Ireland. It is also the first time that *emm* subtype 18.12 has been detected in the country. Rapid delivery of IPC support and chemoprophylaxis were the key outbreak control measures instituted by the OCT, with no further cases linked to the outbreak following the implementation of these measures. There is an ongoing need to reinforce the importance of IPC measures such as hand hygiene, BBE, PPE and appropriate cleaning of shared equipment including electronic devices in all clinical environments. Given the increased mortality among nursing home residents with iGAS, Irish national guidance should be expanded to include management of cases and outbreaks in nursing homes. In the absence of national guidelines, our use of the UKHSA guidelines shows the value of open, visible sharing of guidance and best practices from all relevant actors nationally and internationally. *Emm*18 is infrequently associated with GAS infections in Ireland and elsewhere.
